# Quality of screening with conventional Pap smear in Austria – a longitudinal evaluation

**DOI:** 10.1186/1471-2458-13-998

**Published:** 2013-10-23

**Authors:** Éva Rásky, Peter Regitnig, Michél Schenouda, Nathalie Burkert, Wolfgang Freidl

**Affiliations:** 1 Institute of Social Medicine and Epidemiology, Medical University Graz, Universitaetsstrasse 6/I, 8010 Graz, Austria; 2 Institute of Pathology, Medical University Graz, Auenbruggerplatz 25, 8036 Graz, Austria; 3 Austrian Society of Cytology, Auenbruggerplatz 25, 8036 Graz, Austria

**Keywords:** Quality assurance, Pap smear, Screening, Austria, Self-monitoring

## Abstract

**Background:**

In recent decades, the incidence of cervical cancer and cervical cancer mortality in Austria has declined by varying degrees. The Pap smear is to be considered a causal factor for this decline.

**Methods:**

This longitudinal analysis is based on a data set of Pap smear assessments collected by the Committee for Quality Assurance of the Austrian Society of Cytology. Data from 15 laboratories participating in a voluntary self-monitoring program was analyzed for the time span 2004–2008. The data was analyzed in terms of smear quality and assessment quality.

A rank-correlation-test for a monotonic trend analysis in the proportion of the three parameters Pap 0, “satisfactory, but limited/SBL”, and Pap IIID/IV for the timespan 2004 to 2008 was carried out.

**Results:**

For this study, we analyzed an average number of 730,000 smears per year over a five-year period. Specimens from all but two laboratories, i.e. < 2% of all smears, met the quality criterion for Pap 0 (Bethesda 2001 equivalent: Specimen processed and examined, but unsatisfactory for evaluation of epithelial abnormality), whilst only four laboratories, i.e. < 10% of all smears, reached the national requirement for smears classified as “satisfactory, but limited/SBL”.

When using the Pap IIID/IV ratio (LSIL: HSIL/AIS ratio) of 3:1 to 8:1 as a surrogate quality marker for the interpretation of smears, only five laboratories met this criterion during the survey period.

The trend analysis indicated only that an increasing number of samples per year is correlated with an increased proportion of Pap 0 and “satisfactory, but limited/SBL” smears.

**Conclusions:**

Although participants get regular feedback about their results, no general improvements in smear taking or assessment were observed over the years, so mandatory quality management, including the possibility of sanctions, is suggested in order to reduce adverse health effects for women.

## Background

In recent decades, the incidence of cervical cancer in the industrialized world, as in Austria, has declined [1]. From 1983 to 2009 the age-standardized incidence declined by 65%, from 19.2 to 6.6 per 100,000 women; in absolute numbers: from 954 to 394 women [2]. During the same period, the specific mortality rate dropped by 54% (from 4.4 to 2.0 per 100,000 women; in absolute numbers: from 265 to 141 women). For women aged up to 75, the life-time prevalence of contracting the disease is 1.9%, and the risk of dying from it is 0.5% [1]. Between 1980 and 2010 the cumulative likelihood for women aged 15 to 79 of developing cervical cancer dropped from 2.8% (1.3-2.6 [95% confidence interval]) to 1.0% (0.7-1.6 [95% confidence interval]) [3]. Although a diagnosis of cervical cancer puts enormous strain on the women affected, cervical cancer from a public health perspective is actually not considered to be a major risk for the female population at large, not least because primary and secondary preventive measures are available.

The Austrian Federal Ministry of Health recommends, as a primary preventive measure, the vaccination of both girls and boys aged between 9 and 12 years against Human Papillomavirus/HPV [4]. In most Austrian provinces, the costs for HPV vaccination are borne by the consumer, i.e. generally the parents. Therefore, the vaccination rate in Austria is only approximately 2–5%, although it varies from province to province. Experts agree that early detection measures will still be necessary even after individual HPV vaccination. For this purpose, smear tests according to Papanicolaou will remain an important tool. Although the effectiveness of conventional cytology for cancer screening has never been tested in randomized studies, the results of cohort studies are considered to be sufficient proof [5]. Screening should discover dysplasia at an early stage and with the subsequent interventions morbidity and specific mortality rates will be reduced.

Austria has been providing Pap smear testing to women through opportunistic screening since the 1970s [6,7]. Austrian medical societies recommend an annual smear test for all women from the age of 19, as part of their gynecological examination [8]. The costs for these examinations are borne by the statutory health insurance as part of the “new prevention program” regardless of individual insurance coverage. The Pap smears are mainly taken by gynecologists in their offices. The taking and assessment of smears is mostly done using the “conventional” rather than the “liquid-based” method, because the latter is not covered by health insurance providers. HPV testing is funded by statutory health insurances only in certain specified cases, varying from province to province.

Since the introduction of cancer early detection programs and in particular since the European Commission Directive 2003/878/RG regarding screening, quality assuring measures have been widely discussed throughout Europe. The European Union drafted guidelines on quality assurance [9-11] for screening procedures which, according to the EU, provide demonstrable benefits. Members of the European Parliament signed a resolution that the “fight against cancer” should include program screening [12].

In Austria, the Guideline of the European Commission recommending program screening with centralized monitoring has so far not been implemented [10,13]. Both the Austrian Society of Cytology and the Austrian Society of Gynecology and Obstetrics recommend tools for smear taking and assessment in accordance with the European Guidelines [14,15]. However, a continuous systematic quality control is lacking [7]. Only a small number of scientific articles have hitherto studied the quality of opportunistic screening in Austria. These publications identified failures in Pap smear taking as well as in the interpretation of the smears [16-18]. The results have led the statutory health insurance providers of some provinces to implement a number of measures in order to improve Pap smear taking [19,20]. Furthermore, the Quality Assurance Committee of the Austrian Society of Cytology initiated a database for cytology results commencing in 1998. This initiative aims to improve screening quality [21]. Evaluating the data sets allows a yearly benchmarking for the participating laboratories based on their data concerning Pap smear taking and interpretation. The guidelines of the Austrian Society of Cytology require that the laboratories give gynecologists regular quality feedback. Each gynecologist submitting more than 100 smears annually for testing should receive a report on the smears taken, comparing them with the anonymized list of all smear takers using the cytological laboratory. The reason for introducing this inclusion criterion of a minimum of 100 smears is to reduce statistical variability.

The database of the Austrian Society of Cytology provides the basis for our first longitudinal analysis of Austrian data. Evaluating the quality of Pap smear taking and interpretation is important in order to ensure that women receive reliable results regarding cervical lesions. In addition, evaluation of the present opportunistic screening provides baseline data for a program screening in the future. The longitudinal analysis also allows us to assess the quality trend over the years. Without improvements in quality including systematically collecting data the targeted reduction in cervix cancer morbidity and mortality can not be achieved.

## Methods

The Quality Assurance Committee of the Austrian Society of Cytology has been gathering data on Pap smear taking and assessment since 1998. All cytological laboratories in Austria are invited to participate in the program. The participating cytological laboratories report their data on a voluntary basis. Although the number of participating laboratories increased in recent years, not all laboratories participate in this voluntary self-monitoring program. Currently 35 laboratories, i.e. approximately 80% of all Austrian laboratories, take part [22].

The anonymized data set allow the evaluation of Pap smear taking and interpretation over an extended period of time. For our analysis we chose a period of five years: 2004 to 2008. Data for this period existed for 15 (covering 0.73 million smears) of the 35 participating laboratories (in total 1.03 to 1.65 million smears). These 15 laboratories reported their results annually for at least four years in the chosen period. This study covers laboratories that appraised more than 10,000 screening tests each per year.

Pap classification and classification of smear quality was done in accordance with the national guidelines of the Austrian Society of Cytology (see the Additional file 1). Smear quality is given as i) satisfactory, ii) satisfactory, but limited/SBL, or iii) Pap 0 – unsatisfactory. All three categories are defined in detail by the Austrian Society of Cytology [15]. Despite reduced smear quality, e.g. a lack of endocervical cells or a moderately reduced number of squamous cells, the second category leaves room for Pap classification. Although the Austrian quality categories are similar to the Bethesda 2001 classification, a one-to-one conversion to the Bethesda 2001 categories for smear adequacy is not possible.

As an indicator for the quality of smears, we selected the proportion of all specimens sent in and those assessed as i) Pap 0 (Bethesda 2001 best fitting equivalent: Specimen processed and examined, but unsatisfactory for evaluation of epithelial abnormality) or ii) “satisfactory, but limited/SBL”. This indicator is strongly dependent on the quality of smear taking and is therefore strongly dependent on the gynecologist taking the smear. On the other hand, the interpretation of the cytological features with the well-known intra- and interobserver variability is dependent on the cytologist, even though variability can be minimized by using detailed definitions of smear adequacy interpretation [20]. The quality standard for the indicator as set by the Austrian Society of Cytology provides for a maximum of 2% Pap 0 classifications (Bethesda 2001 equivalent: Specimen processed and examined, but unsatisfactory for evaluation of epithelial abnormality) in relation to all smears taken [15]. The national standard, also set by the Austrian Society of Cytology, requires that the category “satisfactory, but limited/SBL” should apply to less than 10% of all smears taken. Quality improvement was assumed to have taken place if the number of both classifications decreased over the survey period. The assumption being that if appropriate information is given, if smear takers and cytological appraisers communicate with each other, then improvement can take place.

In order to evaluate smear interpretation quality we chose a specific Pap IIID/IV ratio (LSIL : HSIL/AIS ratio) as quality indicator. It provides a simple surrogate parameter for morphologic interpretation, classifying dysplastic cells as either Pap IIID (LSIL) or Pap IV (HSIL/AIS). This indicator is strongly dependent on the interpretation of the cytomorphological features and on the age of the screened population. This ratio is higher for women under 25 years of age. Although age is a strong confounding factor with regard to this ratio, we assume that the age distribution of women, whose smears were taken, is rather similar across the participating laboratories. For this purpose we postulated a benchmark of 3:1 to 8:1, which corresponds to the anticipated natural distribution of low-grade to high-grade cervical intraepithelial neoplasm/CIN of 4:1 [23]. About 20% of the CIN1 cases progress to CIN2/3, whilst 80% of all cytologically detected CINs ought to fall into group Pap IIID and 20% into group Pap IV [24-26]. In other words, a ratio of 3:1 implies that 75% of all cytologically detected CINs are classified as Pap IIID (LSIL), and a ratio of 8:1 implies that 89% are classified as Pap IIID (LSIL). A German study found Pap IIID (LSIL) in 1.05% of the smears and Pap IV (HSIL) in 0.135%, which corresponds to a ratio of 8,8:1 [23]. The central point to realize is reducing the number of CIN2/3 in screened populations. In this light, a higher ratio seems less problematic. A ratio higher than 8:1 or lower than 3:1 could point to interpretation errors. A limitation of this ratio (Pap IIID/IV) (LSIL : HSIL/AIS) is that CIN2 is assignable to both the Pap IIID group (LSIL) and the Pap IV group (HSIL/AIS), depending on the amount of CIN2 cells found in relation to low-grade dysplastic cells.

We also carried out a rank-correlation-test in order to test for a monotonic trend in the proportion of the parameters Pap 0, “satisfactory, but limited/SBL”, and Pap IIID/IV for the timespan 2004 to 2008 [27]. Spearman’s rank correlation was performed between these parameters and the year of evaluation, separately for each laboratory. A positive correlation indicates an increase in the proportion over the five years, whereas a negative coefficient indicates a decrease. In order to aggregate the findings of the 15 laboratories, summary statistics (minimum, maximum, median, and inter quartile-range) and a meta-analysis of the rank-correlation coefficients according to the random effect model were performed [28]. In this meta-analysis, the mean correlation coefficient of the 15 laboratories and a test for homogeneity among the correlations were calculated. A statistically significant p-value in this homogeneity test indicates that the correlations obtained from the different laboratories differ in magnitude. Because the number of samples analyzed in the laboratories differed from laboratory to laboratory and from year to year, we also tested for a possible relation between the proportions of the parameters Pap 0, “satisfactory, but limited/SBL”, and Pap IIID/IV and the number of samples analyzed per year for the timespan 2004 to 2008. Spearman’s rank correlation was performed separately for each laboratory and then aggregated as described above. Finally, a partial correlation between the proportions of the parameters Pap 0, “satisfactory, but limited/SBL”, and Pap IIID/IV and the year of evaluation, was carried out in order to test for a monotonic trend from 2004 to 2008 while controlling for the number of samples.

## Results

In Austria, with an overall population of 8.4 million, the potential target population (women over 20 years of age) is 3.48 million [29]. In the period from 2004 to 2008 gynecologists took an estimated average of 2 to 2.2 million Pap smears per year (personal information from the Main Association of Austrian Social Security Institutions). These Austrian figures are, however, not precise because a large number of smears are taken in private gynecologists’ offices and are thus not counted by the statutory health insurance. Overall, the 15 selected laboratories included in this analysis appraised a total of 730,000 Pap smears on average per year (Table 1), representing approximately one third of all Pap smears taken in Austria.

**Table 1:** Number of Pap smears per laboratory* and year

	**2004**	**2005**	**2006**	**2007**	**2008**	**Total**
	17,224	10,175	10,941	19,061	17,222	74,623
	16,010	15,390	15,318	15,975	14,082	76,775
	**	18,282	18,637	19,488	21,655	78,062
	16,627	18,258	17,727	17,538	17,542	87,692
	**	19,085	22,657	24,607	24,618	90,967
	**	10,492	30,150	32,082	31,394	104,118
	20,065	20,360	23,829	29,224	33,365	126,843
	43,177	40,527	36,546	10,475	**	130,725
	**	47,057	45,095	47,383	48,345	187,880
	50,018	47,674	47,672	50,716	56,807	252,887
	59,511	59,804	62,862	64,043	65,524	311,744
	90,994	95,070	93,233	98,439	**	377,736
	77,712	74,627	74,632	80,108	72,605	379,684
	103,403	102,480	102,437	104,032	104,728	517,080
	175,171	172,457	170,132	168,697	164,656	851,113
Total	669,912	751,738	771,868	781,868	672,543	3,647,929

*Laboratories were identified by the number of specimens submitted. To safeguard anonymity the laboratories in Table 1 have no abbreviation, and the sequence of the laboratories in Tables 1 and 2 has been changed. Despite the loss of relevant information, this approach was necessary since non-anonymity might have discouraged laboratories from participating.

**no data available.

The number of specimens entered per laboratory ranges from somewhat more than 10,000 to almost 200,000 per year. Certain individual laboratories had large changes in the number of smears submitted over the 5-year period, although half of the laboratories had stable numbers (Table 1).

Table 2 presents the percentage of all Pap smears sent to the laboratory and assessed as Pap 0 (“Specimen processed and examined, but unsatisfactory for evaluation of epithelial abnormality”). All in all, only two out of 15 laboratories failed to meet the quality standards [15]. Unlike the Bethesda classification, the Austrian Pap classification entails remarks on minor quality deficiencies in an own smear quality category as “satisfactory, but limited” (SBL). Smears assessed as SBL still allow Pap classification. However, a false negative result is more likely in such case, compared to smears assessed as “satisfactory” [30-32]. When considering the proportion of specimens that were classified as SBL a different picture emerges (Table 3). Only four out of 15 laboratories were actually below the required 10% limit concerning their annual results across all smears submitted by gynecologists in the survey period. When considering only those laboratories that supplied data throughout the entire survey period, only one (out of six) complied with the national standard. Over the whole period, two of the laboratories had even more than 30% of their smears classified as SBL. The results of the trend analysis for the period 2004 to 2008 showed that no conclusion can be reached about a positive or negative trend in relation to lower or higher proportions of Pap 0 and SBL categories. This is due to the heterogeneity of laboratory sample sizes and the proportion of Pap 0 and SBL. Nonetheless, we assumed that the recommended feedback of cytologists to gynecologists regarding a possible modification of their smear taking practice has no influence on quality improvement.

**Table 2:** Percentage of all Pap smears submitted to a laboratory* that were assessed as Pap 0^**+ **^**(Bethesda 2001 equivalent: specimen processed and examined, but unsatisfactory for evaluation of epithelial abnormality)**

	**2004**	**2005**	**2006**	**2007**	**2008**
Lab A	0.17	0.19	0.26	0.29	0.0
Lab B	0.21	0.29	0.15	0.26	0.21
Lab C	0.10	0.10	0.07	0.11	0.09
Lab D	**	0.91	1.08	1.03	0.67
Lab E	0.48	0.45	0.51	0.76	0.91
Lab F	0.17	0.18	0.23	0.17	**
Lab G	**	**4.13**	**2.35**	**3.74**	**2.61**
Lab H	0.67	0.58	0.50	0.58	1.25
Lab I	0.63	0.65	0.70	0.65	0.09
Lab J	1.62	**3.68**	**3.66**	**2.21**	1.95
Lab K	0.16	0.17	0.69	0.67	0.95
Lab L	0.18	0.26	0.26	0.35	0.16
Lab M	0.07	0.00	0.12	0.07	0.12
Lab N	**	0.02	0.30	0.46	0.40
Lab O	**	0.42	0.43	0.36	0.48

*Laboratories were identified by the number of specimens submitted. To safeguard anonymity the laboratories in Table 1 have no abbreviation, and the sequence of the laboratories in Tables 1 and 2 has been changed. Despite the loss of relevant information, this approach was necessary since non-anonymity might have discouraged laboratories from participating.

^+^A national standard, set by the Austrian Society of Cytology, requires that the category Pap 0 should be less than 2% of all smears taken (15).

**no data available;

Laboratories failing to meet the quality criterion are written in bold.

**Table 3:** Percentage of all smears submitted to a laboratory* that were assessed as “satisfactory, but limited/SBL”^**+**^

	**2004**	**2005**	**2006**	**2007**	**2008**
Lab A	**22.94**	**22.04**	**21.17**	**17.50**	**
Lab B	**18.76**	**	**14.02**	**	**13.37**
Lab C	6.76	8.04	**10.90**	7.86	7.55
Lab D	**	1.28	2.95	9.08	7.45
Lab E	**50.02**	**54.07**	**48.35**	**52.69**	**46.04**
Lab F	5.73	6.90	**11.59**	9.81	**
Lab G	**	**26.15**	**29.51**	**28.86**	**25.00**
Lab H	2.39	6.40	7.64	9.05	**13.66**
Lab I	**21.09**	**21.61**	**21.59**	**	**13.46**
Lab J	**68.51**	**45.78**	**35.70**	**45.40**	**38.94**
Lab K	4.89	5.49	4.85	4.68	5.57
Lab L	**28.96**	**24.26**	**25.12**	**26.61**	**27.04**
Lab M	**	**	**16.74**	7.81	7.27
Lab N	**	2.16	6.55	9.52	9.24
Lab O	**	2.12	5.05	5.89	7.01

*Laboratories were identified by the number of specimens submitted. To safeguard anonymity the laboratories in Table 1 have no abbreviation, and the sequence of the laboratories in Tables 1 and 3 has been changed. Despite the loss of relevant information, this approach was necessary since non-anonymity might have discouraged laboratories from participating.

^+^A national standard, set by the Austrian Society of Cytology, requires that the category “satisfactory, but limited/SBL” should be less than 10% of all smears taken (15).

**no data available;

Laboratories failing to meet the quality criterion are written in bold.

Table 4 presents the Pap IIID/IV ratios (LSIL : HSIL/AIS ratios) of the specimens sent in to the laboratories. The surrogate interpretation quality indicator, the Pap IIID/IV ratio (LSIL: HSIL/AIS ratio) was achieved by five laboratories. For every given Pap IV (HSIL/AIS), more than twice the number of Pap IIID (LSIL) was found.

**Table 4:** Pap IIID/IV ratio (LSIL : HSIL/AIS ratio) and absolute numbers of Pap IIID and IV smears (in brackets)

	**2004**	**2005**	**2006**	**2007**	**2008**
Lab A	8.76*	5.01	*8.57*	5.33	**
	*(394/45)*	(361/72)	*(317/37)*	(128/24)	
Lab B	*15.09*	*12.52*	*10.16*	*10.56*	*11.90*
	*(332/22)*	*(288/23)*	*(315/31)*	*(338/32)*	*(595/50)*
Lab C	3.85	4.34	6.22	5.23	5.38
	(1096/285)	(1185/273)	(970/156)	(904/173)	(1087/202)
Lab D	**	3.81	7.75	6.10	7.83
		(118/31)	(248/32)	(354/58)	(360/46)
Lab E	7.86	*13.60*	*8.88*	*9.75*	4.07
	(55/7)	*(68/5)*	*(71/8)*	*(78/8)*	(57/14)
Lab F	4.24	4.48	5.63	5.20	**
	(704/166)	(695/155)	(676/120)	(567/109)	
Lab G	**	3.36	5.74	4.38	5.66
		(131/39)	(402/70)	(254/58)	(362/64)
Lab H	*16.13*	*12.68*	*13.56*	*10.23*	5.17
	*(1145/71)*	*(926/73)*	*(1085/80)*	*(982/96)*	(703/136)
Lab I	**2.23**	3.08	4.33	4.46	3.72
	**(1439/644)**	(1934/628)	(1952/451)	(1747/392)	(1494/402)
Lab J	3.13	**0.69**	**0.51**	**1.67**	**2.56**
	(94/30)	**(22/32)**	**(18/35)**	**(87/52)**	**(105/41)**
Lab K	**2.58**	**1.91**	3.18	**2.59**	3.48
	**(170/66)**	**(124/65)**	(226/71)	**(275/106)**	(432/124)
Lab L	*9.74*	5.60	7.00	7.63	6.87
	*(380/39)*	(454/81)	(441/63)	(473/62)	(364/53)
Lab M	6.45	3.61	4.37	4.11	5.92
	(71/11)	(101/28)	(131/30)	(115/28)	(142/24)
Lab N	**	*10.60*	*17.51*	*14.89*	*13.80*
		*(1421/134)*	*(1173/67)*	*(1385/93)*	*(1270/92)*
Lab O	**	*9.87*	4.73	*21.04*	*13.75*
		*(464/47)*	(430/91)	*(484/23)*	*(495/36)*

* read as ratio to 1 for every figure;

** no data available;

The ratios of laboratories falling below the ratio of 3:1 are written in bold.

The ratios of laboratories exceeding the ratio of 8:1 are written in italics.

The summary statistics and the test for homogeneity of the correlations show, in most cases, a great variation in trend between the 15 laboratories (Additional file 1). The averaged correlation between the proportion of all the three parameters of interest and the year of observation was rather low (Spearman’s rho ranged from 0.12 to 0.35). There was also little change in magnitude of the correlation after controlling for the number of samples. The highest averaged correlations were found between the proportion of the parameter and the number of samples analyzed per year for Pap 0 (rho = 0.56) and for the “satisfactory, but limited/SBL” (rho = 0.48), indicating that the proportion of Pap 0 and of the “satisfactory, but limited/SBL” rose as the number of samples analyzed per year increased. However, a statistically significant deviation from homogeneity was found for both parameters, indicating a substantial variation between the laboratories.

## Discussion

The purpose of this analysis was to assess the quality of opportunistic screening by evaluating a nationwide data set. In Austria, only a small number of studies on the quality of opportunistic Pap screening have been conducted so far [16-18]. Our study shows again that failures in Pap smear taking and interpretation of smears exist. We therefore emphasize the relevance of regular feedback and systematic data collection, monitoring and evaluation to improve the quality of Pap screening. Without regular, systematic and mandatory quality checks adverse effects of screening on women cannot be assessed. Reducing further cervical cancer morbidity and mortality will be impossible.

The validity of this study is limited by the fact that not all Austrian laboratories participate in this voluntary self-monitoring program initiated and implemented by the Austrian Society of Cytology. Another limiting factor for the validity of this paper is that only 15 out of the 35 participating laboratories regularly reported data in the study period, meaning they provided data for at least four annual reports and were thus included in this evaluation. This low level of participation and reporting can be partly explained by the fact that reporting requires specific resources and IT support in the laboratories.

Even when accounting for these limitations, the high proportion of Pap smears assessed as “satisfactory, but limited/SBL” is particularly alarming. Heterogeneity of sample sizes across the laboratories and proportions of Pap 0 and SBL categories during the study period limited the conclusiveness of the study performed. Trend analysis only showed that laboratories carrying out a higher number of smear tests had higher proportions of Pap 0 and SBL smears, meaning a larger number of gynecologists failed to meet the quality requirements.

Since the data set is based on voluntary reporting, the actual number of failures may even be higher than shown in this evaluation. These deficiencies need to be corrected as soon as possible. Decision-makers are advised in the strongest possible terms to take action. In order to assess and eventually eliminate existing quality deficiencies, mandatory reporting seems to be an absolute necessity. Statutory health insurance providers should by any means ensure that their contractual partners (gynecologists and laboratories) sign a binding agreement concerning their active involvement in such quality assurance measures. The best option to start with would be rolling out a tried and tested model quality assurance project in all Austrian provinces [19,20]. Workshops on Pap smear quality and Pap smear taking practice performed in the past effectively lowered “satisfactory, but limited/SBL” rates over longer time periods [19].

Given that the surrogate ratio has limitations and that we have no information on the age of the women of whom the Pap smears have been taken, deficiencies in the interpretation of the Pap smear results are evident. Regarding the IIID/IV ratio (LSIL : HSIL/AIS ratio) under these premises, our results show shortcomings across the whole survey period. To enhance assessment quality, health professionals should be given advanced training. An additional option would be to establish mandatory external audits for all cytological laboratories evaluating smear tests. Their participation in monitoring processes should be remunerated accordingly.

Reimbursing only those services that meet the quality standards and enhancing continuous and continuing education of the service providers will not suffice to ensure that the European Guidelines are met. These guidelines recommend program screening which includes the definition of a target population, a standardized approach to smear taking and subsequent diagnostic procedures, quality standards for the interpretation of smears and monitoring of the entire screening process, e.g. data collection and analysis. Competing interests of stakeholders in the field impede the establishing of a standardized program for cervical cancer screening. Given the failures in the present opportunistic screening, there is an urgent need for action in order to rapidly improve Pap smear taking in the short term. The longitudinal evaluation shows that feedback from cytologists to gynecologists regarding smear taking quality and benchmarking of gynecologists’ smear results on a voluntary basis have not led to changes in professional behavior. The voluntary self-monitoring program in Austria has proven to be insufficient when it comes to improving quality. There have been no appreciable improvements in any of the measures over the time period studied and at any individual laboratory. Therefore, monitoring should take place on a mandatory basis. This seems to be crucial since due to the low uptake of a population-wide HPV vaccination Austria currently has no cohorts of lower-risk young women.

## Conclusions

In conclusion and despite the limitations of this study, we found strong indications concerning the shortcomings of conventional Pap screening in Austria, in Pap smear taking, assessment, and in gathering relevant data. It was beyond the scope of this study to assess how many women have been harmed by these failures. The real extent of the deficiencies will only become visible upon introducing mandatory participation for all laboratories and gynecologists. Sanctions for non-compliant laboratories and gynecologists are necessary to enforce their adhesion to the quality assurance process. In particular health insurance providers and the Federal Ministry of Health are called upon, for example to develop quality indicators and implement effective controlling. It is essential that the Austrian Federal Minister of Health decree a legally binding federal quality guideline as the most effective means of addressing the concerns raised in this study. This standard should also cover the potential for a future change in cervical screening test from cytology to a primary HPV test [33-35]. A further delay in taking action is harming women with overdiagnosis and overtreatment, or by detecting cervical cancer at a late stage.

### Ethics committee

No voting required, secondary analysis of anonymized data.

## Competing interests

The authors declare that they have no competing interests.

## Authors’ contributions

ER conceptualizing, designing, interpreting of data and drafting the article; PR conceptualizing, designing the article, acquisitioning of data, analyzing data; MS analyzing data; NB revising the article for important intellectual content; WF designing data analysis, revising manuscript critically for important intellectual content. All authors read and approved the final manuscript.

## Pre-publication history

The pre-publication history for this paper can be accessed here:

http://www.biomedcentral.com/1471-2458/13/998/prepub

## Supplementary Material

Additional file 1 Pap classification by the Austria Society of Cytology in correlation with the Bethesda classification. Click here for file
