# Supplementary material for: Quality of screening with conventional Pap smear in Austria – a longitudinal evaluation
Source: BMC Public Health. 2013 Oct 23;13:998. doi: 10.1186/1471-2458-13-998 (PMC4015555; doi:10.1186/1471-2458-13-998)
Supplement: Additional file 1 — Pap classification by the Austria Society of Cytology in correlation with the Bethesda classification. [file 1471-2458-13-998-S1.doc]

**Pap classification by the Austria Society of Cytology in correlation with the Bethesda classification**

**Smear quality (abridged)**

1. Satisfactory
   1. Satisfactory and representative (e.g. 8,000 – 10,000 squamous cells; endocervical cells or metaplastic cells present)
   2. Satisfactory, but limited (e.g. absence of endocervical cells and or metaplastic cells; reduced number of squamous cells; obscuring blood or inflammatory cells, other reasons)
2. Unsatisfactory (Pap 0)

| **Pap** | Austrian Pap Classification 2005 | Bethesda 2001 Equivalent |
| --- | --- | --- |
| 0 | Not assessable   - not processed because of technical/ administrative shortcomings - processed – but not assessable because… | Specimen processed and examined, but unsatisfactory for evaluation of epithelial abnormality  • Specimen rejected/not processed  (specify reason) |
| I* | Normal, age-typical cell profile in representative smears; light inflammation without epithelial change; metaplasia |  |
| II* | Inflammatory, regenerative metaplastic or degenerative change; typical endometrium cells (specification “post-menopausal” compulsory). Hyper- and Parakeratosis; HPV-associated lesion without suspicious cell nucleus alteration; atrophic cell profile with autolysis | Negative for epithelial lesion or  malignancy/other; LSIL (only  HPV) |
| III | Higher-grade inflammatory and/or degenerative and/or atrophic lesion of undetermined significance (CIN or invasive carcinoma not excluded) | ASC-US; ASC-H |
| IIID | Cells showing low- to medium-grade dysplasia (CIN I-II) | LSIL |
| IIIG | Atypical cervical or endometrial glandular cells (suspicion of proliferous or neoplastic lesions) | Atypical endocervical, endometrial,  glandular (NOS) cells,  atypical endocervical or glandular  cells, favor neoplastic |
| IV | Cells showing medium- to high-grade dysplasia or squamous epithelium or adenocarcinoma in situ (CIN II-III, AIS). No tangible clue to invasion. | HSIL (without or with features  suspicious for invasion), endocervical  adenocarcinoma in situ  (AIS) |
| V | Cells indicating presumptive squamous epithelia or adenocarcinoma of the cervix or other malignant tumors. | Squamous cell carcinoma,  adenocarcinoma (endocervical,  endometrial, extrauterine,  NOS); other malignant neoplasms |

* Pap I and II may be grouped as one category, Pap II

ASCUS = atypical squamous cells of undetermined significance

LSIL = low-grade squamous intraepithelial lesion (= low-grade dysplasia and HPV infection)

HSIL = high-grade squamous intraepithelial lesion (medium- and high-grade dysplasia, carcinoma in situ)

Table A: Monotonic trend analysis of changes in the proportion of PAP 0, “satisfactory, but limited/SBL”, and PAP IIID/IV from 2004 to 2008: Summary Statistics for 15 Austrian laboratories

|  | Spearman's rho between parameter and year | Spearman's rho between parameter and number of samples | Partial correlation between parameter and year controlling for the number of samples |
| --- | --- | --- | --- |
| *Pap 0* |  |  |  |
| Minimum | -0.40 | -1.00 | -0.60 |
| 25th percentile | -0.30 | -0.30 | -0.36 |
| Median | 0.00 | 0.60 | 0.20 |
| 75th percentile | 0.80 | 1.00 | 0.50 |
| Maximum | 1.00 | 1.00 | 0.89 |
| Meta analysis: |  |  |  |
| Mean of rho | 0.35 | 0.56** | 0.16 |
| Homogeneity test: *p* | 0.253 | <0.001 | 0.641 |
| *“satisfactory, but limited/SBL”* |  |  |  |
| Minimum | -1.00 | -1.00 | -1.00 |
| 25th percentile | -0.70 | -0.30 | -0.72 |
| Median | 0.00 | 0.60 | -0.14 |
| 75th percentile | 0.80 | 1.00 | 0.89 |
| Maximum | 1.00 | 1.00 | 1.00 |
| Meta analysis: |  |  |  |
| Mean of rho | 0.31 | 0.48* | 0.47 |
| Homogeneity test: *p* | <0.001 | <0.001 | <0.001 |
| *Pap IIID/IV* |  |  |  |
| Minimum | -0.90 | -1.00 | -0.91 |
| 25th percentile | -0.30 | -0.60 | -0.75 |
| Median | 0.20 | 0.40 | -0.23 |
| 75th percentile | 0.70 | 0.70 | 0.74 |
| Maximum | 0.80 | 0.90 | 0.90 |
| Meta analysis: |  |  |  |
| Mean of rho | 0.12 | -0.04 | -0.19 |
| Homogeneity test: *p* | 0.441 | 0.002 | 0.072 |

Note: Spearman's rho between parameter and year denotes the correlation between the proportion of the parameter of interest and the year of evaluation (2004 to 2008).

Spearman's rho between parameter and number of samples denotes the correlation between the proportion of the parameter of interest and the number of samples analyzed in the laboratories across the years from 2004 to 2008.

Meta analysis was carried out according to the random-effect model.

* *p* < 0.05, ** *p* < 0.01.
